# 3D printed dummy heads for crosstalk cancellation studies in bone conduction

**DOI:** 10.1016/j.ohx.2024.e00618

**Published:** 2024-12-25

**Authors:** Irwansyah, Sho Otsuka, Seiji Nakagawa

**Affiliations:** a Center for Frontier Medical Engineering, Chiba University, Chiba, Japan; b Graduate School of Engineering, Chiba University, Chiba, Japan; c Med-Tech Link Center, Chiba University, Chiba, Japan

**Keywords:** 3D printing, Dummy heads, Bone conduction, Crosstalk cancellation, Silicone skins, Vibration analysis

## Abstract

Thanks to affordable 3D printers, creating complex designs like anatomically accurate dummy heads is now accessible. This study introduces dummy heads with 3D-printed skulls and silicone skins to explore crosstalk cancellation in bone conduction (BC). Crosstalk occurs when BC sounds from a transducer on one side of the head reach the cochlea on the opposite side. This can disrupt binaural cues essential for sound localization and speech understanding in noise for individuals using BC hearing devices. We provide a step-by-step guide to constructing the dummy head and demonstrate its application in canceling crosstalk. The 3D models used in this study are freely available for replication and further research. Several dummy heads were 3D-printed using ABS for the skull and silicone skins of varying hardness, with a 3-axis accelerometer at the cochlea location to simulate inner ear response. Since the cochlea is inaccessible in humans, we targeted crosstalk cancellation at the mastoid, assessing if this cancellation extended to the cochlea within the dummy heads. We compared these results with our previous experiments conducted on seven human subjects, who had their hearing thresholds measured with and without crosstalk cancellation, to evaluate if the dummy heads could reliably replicate human crosstalk cancellation effects*.*

## Graphical abstract

**Figure:**
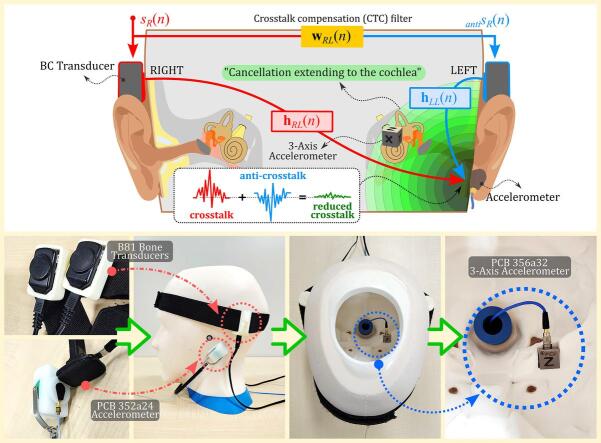

**Specifications table**

**Table:** 

Hardware name	Anatomically Correct 3D-Printed Skulls with Silicone Skins
Subject area	• Engineering and materials science • Medical • Educational tools and open-source alternatives to existing infrastructure
Hardware type	• Measuring physical properties and in-lab sensors • Mechanical engineering and materials science • Anatomical modeling and acoustic measurement tools for bone conduction research
Closest commercial analog	“*No commercial analog is available*”
Open-source license	CC-BY-SA-4.0 (Creative Commons Attribution Share Alike 4.0)*.*
Cost of hardware	*∼*$156.43 USD (per dummy head, excluding sensors)
Source file repository	https://doi.org/10.5281/zenodo.13777788

## 1 Hardware in context

### 1.1 Limitations of artificial heads

In acoustic research, artificial heads are commonly used for binaural recordings [1], [2]. These devices are intended to simulate the human head, torso, and ears where microphones are placed in the ear canals to record sound as a person would naturally hear it. This setup ensures a very realistic audio experience. However, using these artificial heads for bone conduction (BC) studies can be complicated. They lack the complex anatomical structures of the skull required to accurately replicate BC sound propagation. Instead, researchers utilize commercial devices designed to mimic the mechanical properties of the human mastoid bone and skull, known as an “artificial mastoid” [3] and a “skull simulator” [4]. Unlike artificial heads, these devices do not resemble human anatomy and are typically used to test one BC transducer at a time. For this reason, they are not suitable for simulating BC scenarios.

### 1.2 Crosstalk in BC: challenges and approaches

**Fig. 1:**
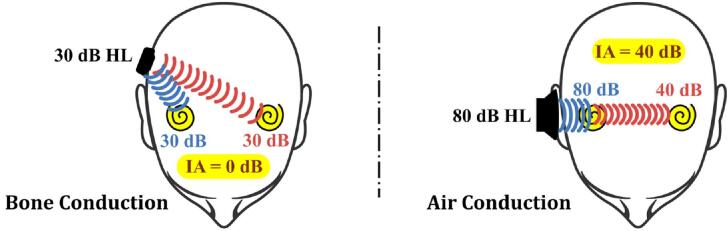
The left panel illustrates crosstalk via BC, resulting in minimal IA. In contrast, the right panel shows AC via insert earphones, where significant IA occurs, reducing sound levels as it crosses to the opposite side.

BC is an alternative means of sound transmission, particularly beneficial for individuals with conductive hearing loss [5]. BC sounds or vibrations generated by a BC transducer attached to the head can be transmitted through the skull directly to the cochlea in the inner ear [5], [6]. However, these vibrations are also transmitted to the cochlea on the opposite ear [7], which we term as “crosstalk” in this study. For air conduction (AC) through insert earphones, an AC sound from one earphone loses much intensity as it crosses the head, a reduction known as interaural attenuation (IA) [8] (see Fig. 1). For BC, however, IA is very low, around 0 dB at frequencies below 1 kHz and ranging from 5 to 20 dB at higher frequencies at the BAHA (Bone-Anchored Hearing Aid) location [9]. This means the crosstalk experiences less reduction in intensity when crossing from one ear to the other. The low IA in bone conduction is why crosstalk sound can be a significant issue, as it can weaken the effectiveness of using a pair of BC devices by disrupting binaural cues, which are crucial for sound localization [10], [11], [12] and speech understanding [13].

Crosstalk cancellation techniques are well-known in acoustics, particularly for binaural sound reproduction [14], [15], [16]. To implement these techniques, two microphones can be placed at the target ears to capture sounds from two loudspeakers and use that information to derive crosstalk compensation (CTC) filters. However, for BC, the target cancellation sites—the cochleae—are inaccessible. As a result, researchers must rely on indirect psychoacoustic-based methods to estimate, cancel, and evaluate crosstalk in human heads [17], [18], [19], [20], [21]. Psychoacoustic experiments, however, mainly depend on human participation, which is time-consuming [21] and also heavily reliant on subjective feedback. Therefore, there is a need for accurate and reliable evaluation, which is where a simulator becomes important for this BC study. A simulator offers a controlled and consistent environment for testing crosstalk cancellation approaches before evaluating these methods in human subjects.

### 1.3 Need for dummy heads in simulating BC crosstalk

To simulate crosstalk scenarios, one approach is to use a dry human skull with an accelerometer placed near the cochlea to detect BC sounds. Researchers have experimented with this method for studying BC in general [22]. While 3D-printed heads exist for acoustic studies [23], models specifically designed for BC remain absent. Using dry human skulls for crosstalk studies involves ethical and legal challenges, as human remains are regulated by laws and guidelines [24]. In addition, different skulls can introduce variability due to differences in shapes and sizes. To address these issues, we propose creating anatomically accurate skulls using 3D printers with ABS material, then covering them with silicone of varying hardness levels. By identifying the right combination of ABS and silicone hardness, we aim to replicate the human head response, achieving consistent and reproducible results, particularly for crosstalk cancellation in BC experiments. The dummy head can provide us with standardization and a controlled testing environment.

## 2 Hardware description

**Fig. 2:**
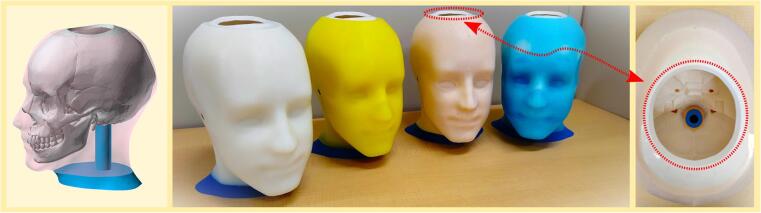
3D model and printed dummy heads. (Left) The 3D model of the modified TARO head model, showing only the skull and face. (Middle) Four printed skulls covered with silicone skins of varying hardness levels. (Right) The open top of the dummy head, designed to allow for the installation of a 3-axis accelerometer.

This paper reports on the development of a dummy head designed to study crosstalk cancellation in bone conduction. The main objective of this study is to demonstrate the potential for an open-access head model in such research. The left panel of Fig. 2 shows the 3D model of the dummy head used in this study. This model has been modified from the original TARO model to include only the skull and face without the neck bones. The TARO model is a 2 mm × 2 mm × 2 mm voxel dataset [25] of a human male, created from full-body magnetic resonance imaging (MRI) by the National Institute of Information and Communications Technology (NICT) [26], and is freely available on the BodyParts3D website under CC-BY-SA 2.1 Japan [27]. This model reproduces the average body type of a Japanese adult and was highly rated by Mata and Sales for its anatomical accuracy and detailed realism [28].

In this study, four skulls were printed and covered with silicone skins of varying hardness levels, from very soft to slightly hard, as shown in the middle panel of Fig. 2. These four dummy heads were evaluated and characterized to simulate crosstalk cancellation scenarios, following the same setup as our previous experiments with human subjects [19]. The results are compared and analyzed to identify which dummy head provides responses similar to those observed in human subjects. In addition, the top of the dummy head is left open with a diameter of approximately 67 mm, as shown in the right panel of Fig. 2, to allow for the installation and re-installation of a 3-axis accelerometer between dummy heads. To summarize, the proposed dummy head has the following features:

• **Anatomically Accurate Design**—Based on MRI scans that reflect the average Japanese adult.
• **Low Cost**—Utilizes 3D printing technology and common materials to keep production affordable.
• **Customizable**—Can be customized and replicated easily, thus making it ideal for experimenting with various materials.

## 3 Design files summary

### 3.1 Design files

The original human model from BodyParts3D includes detailed representations of bones, muscles, vessels, and internal organs, comprising over a thousand individual OBJ files. For this study, we filtered and extracted only the full-body skin, teeth, and skull, which together consist of 51 OBJ files. These files can be manually selected and downloaded from the BodyParts3D website [27]. For convenience, a link to our Zenodo repository (doi:10.5281/zenodo.13777788) is provided, where details about the 51 files are briefly explained, along with a link to download them for creating custom molds for dummy heads if desired. Alternatively, the design files provided in this paper can be used to replicate the dummy head.

To simplify manufacturing, the 51 OBJ files were combined into three STL files representing the skull, jaw, and full-body skin. Minor modifications were made to these original files to adapt them for the study. For example, a hole was added to the top of the skull to allow hand access for placing the accelerometer at the cochlea location. Also, space was created near the cochlea to accommodate the accelerometer. To fit within the printing volume of our 3D printer, the full-body skin model was trimmed around the neck when designing the mold. These final STL files are summarized in Table 1, which also provides the files necessary for the creation of the dummy head:

**Table 1:** Summary of the design files for dummy head and supporting components, including their names, file types, open-source licenses, and locations where they can be accessed.

Design file name	File type	Open-source license	Location of the file
01-SKULL.stl	STL	CC-BY-SA-4.0	https://doi.org/10.5281/zenodo.13777788
02-JAW.stl	STL	CC-BY-SA-4.0	https://doi.org/10.5281/zenodo.13777788
03-MOLD-HALF-1.stl	STL	CC-BY-SA-4.0	https://doi.org/10.5281/zenodo.13777788
03-MOLD-HALF-2.stl	STL	CC-BY-SA-4.0	https://doi.org/10.5281/zenodo.13777788
04-ALIGNMENT-PINS.stl	STL	CC-BY-SA-4.0	https://doi.org/10.5281/zenodo.13777788
05-SKULL-SUPPORT.stl	STL	CC-BY-SA-4.0	https://doi.org/10.5281/zenodo.13777788
06-MOLD-BASE.stl	STL	CC-BY-SA-4.0	https://doi.org/10.5281/zenodo.13777788
07-MOLD-PLUG.stl	STL	CC-BY-SA-4.0	https://doi.org/10.5281/zenodo.13777788
08-SUPPORT-STAND.stl	STL	CC-BY-SA-4.0	https://doi.org/10.5281/zenodo.13777788

**Fig. 3:**
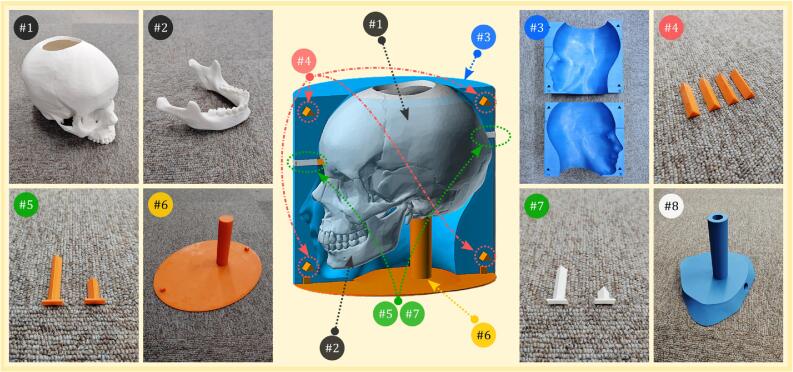
A visual representation of the printed components used in the creation of the dummy head and supporting parts. Each numbered part corresponds to the files listed in Table 1.

Each component in Table 1 is described below and corresponds to a numbered part in Fig. 3, illustrating the 3D printed elements used in constructing the dummy head. Parts #1 and #2 (skull and jaw) are printed in ABS with a 100 % infill, essential for bone conduction. The remaining parts, #3 to #8, are printed in PLA with an infill between 20 − 30 %, as they serve primarily as structural supports:

• **01-SKULL.stl**—(see #1 in Fig. 3)

The main structure of the dummy head, representing the skull.

• **02-JAW.stl**—(see #2 in Fig. 3)

The lower jaw component, designed to fit with the skull.

• **03-MOLD-HALF-1.stl** and **03-MOLD-HALF-2.stl**—(see #3 in Fig. 3)

The two halves of the mold used to shape the silicone skin (or face).

• **04-ALIGNMENT-PINS.stl**—(see #4 in Fig. 3)

Four pins used to align the two halves of the skull mold.

• **05-SKULL-SUPPORT.stl**—(see #5 in Fig. 3)

Two supports used to hold the skull in the center of the mold.

• **06-MOLD-BASE.stl**—(see #6 in Fig. 3)

The base part of the mold used to cover the hole and ensure that the liquid silicone rubber remains inside during curing.

• **07-MOLD-PLUG.stl**—(see #7 in Fig. 3)

Two plug components used to seal the hole in the mold after the initial silicone has cured, ensuring no holes are left on the final product.

• **08-SUPPORT-STAND.stl**—(see #8 in Fig. 3)

A stand designed to support the dummy head during various BC tests.

## 4 Bill of materials summary

### 4.1 Bill of materials

This section contains the bill of materials that was used to build the dummy head. We categorized the materials into three groups: “3D-Printed Parts”, “Consumables”, and “Reusable Items”. The 3D-printed parts include components such as the skull and jaw. Consumables are things we use only once in the construction process, such as liquid silicone rubber, pigments, and other disposable items. Reusable materials, like a digital scale and rubber bands, are used for multiple builds. The costs shown in Table 2 reflect the expenses for making one dummy head, though we actually made four during the validation and characterization phase of this study.

**Table 2:** Bill of materials for constructing a single dummy head.

**Designator**	**Component**	**Number**	Cost per unit-currency*^1^	Total cost-currency*^1^	**Source of materials**	**Material type**
3D-Printed Parts	01-SKULL	977 g	$0.0296	$28.92	jp.store.bambulab	ABS (Polymer)
	02-JAW	91 g	$0.0296	$2.69	jp.store.bambulab	ABS (Polymer)
	03-MOLD-HALF-1	657 g	$0.0296	$19.45	jp.store.bambulab	PLA (Polymer)
	03-MOLD-HALF-2	652 g	$0.0296	$19.30	jp.store.bambulab	PLA (Polymer)
	04-ALIGNMENT-PINS	8 g	$0.0296	$0.24	jp.store.bambulab	PLA (Polymer)
	05-SKULL-SUPPORT	2 g	$0.0296	$0.06	jp.store.bambulab	PLA (Polymer)
	06-MOLD-BASE	106 g	$0.0296	$3.14	jp.store.bambulab	PLA (Polymer)
	07-MOLD-PLUG	2 g	$0.0296	$0.06	jp.store.bambulab	PLA (Polymer)
	08-SUPPORT-STAND	120 g	$0.0296	$3.55	jp.store.bambulab	PLA (Polymer)
Consumables	Liquid Silicone Rubber	2000 g	$0.0322	$64.40	silicreate.com	Polymer
	Silicone Pigment	3 g	$0.3487	$1.05	silicreate.com	Polymer
	Wood Mixing Sticks	10	$0.2007	$2.01	silicreate.com	Organic
	Plastic Cup	10	$0.1560	$1.56	jp.daisonet.com	Polymer
	Tape	1.5 m	$0.0390	$0.06	jp.daisonet.com	Polymer
	Clay	1	$0.1560	$0.15	jp.daisonet.com	Inorganic
Reusable Items	1-kg Digital Scale	1	$9.0141	$9.01	amazon.jp	Composite
	Rubber Band	3	$0.2600	$0.78	jp.daisonet.com	Polymer

1 *Prices were converted to USD using exchange rates of 1 USD = 141 JPY and 1 USD = 1.49 AUD.

For the “3D-Printed Parts”, the listed costs only account for the filament materials and exclude electricity expenses, which can vary based on the 3D printer’s speed settings. In our study, we used the Bambu Lab P1S 3D printer, known for its compatibility with the specified filaments for rapid printing. Due to the complex shapes of the skull and jaw, support structures were required during printing. After removing these supports, the actual weights were approximately 639 g for the skull and 67 g for the jaw, slightly less than the initial weights shown in Table 2. The cost of liquid silicone rubber also varies depending on the hardness level, which impacts the total expense. The costs in Table 2 specifically cover the physical construction of the dummy head, excluding additional equipment like accelerometers, bone transducers, amplifiers, and audio interfaces. These devices are essential for performance validation, particularly in crosstalk cancellation studies, but are not part of the construction process, which is the main focus of this study.

## 5 Build instructions

This section provides detailed step-by-step instructions for creating the dummy head for used in BC studies. We recommend starting with a short video included with the article, which provides a quick overview of the process. The video is also available online at https://youtu.be/5bxazr6eseA.

### 5.1 Printing the components

**Fig. 4:**
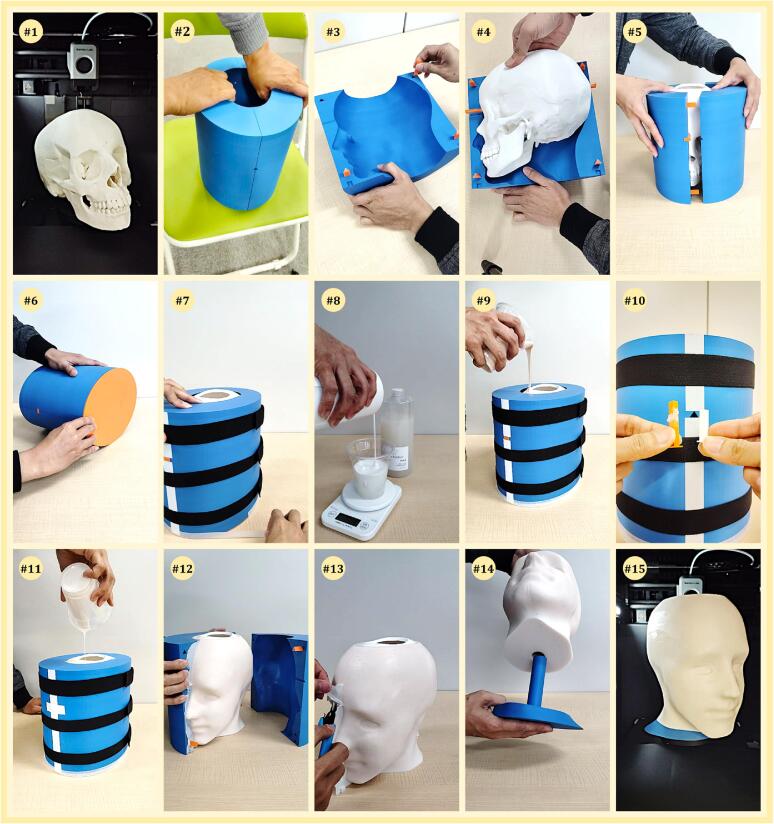
Selected photos of key steps in the dummy head creation. These photos illustrate important stages such as 3D printing components, assembling the mold, and pouring and curing silicone (video: https://youtu.be/5bxazr6eseA).
• **Step 2—**3D print the mold halves (03-MOLD-HALF-1.stl & 03-MOLD-HALF-2.stl) simultaneously using PLA filament with an infill of 20 % to 30 %. Ensure the walls are strong enough by using 4–5 layers; thin walls may break when removing the dummy head with the silicone skin in the final step. When printing both halves together, leave a small 0.4 mm gap between them to make separation easier after printing. As shown in #2 of Fig. 4, use both hands to carefully separate the mold halves after printing.
• **Step 3—**Print the remaining design files. These include the alignment pins (04-ALIGNMENT-PINS.stl), the skull supports (05-SKULL-SUPPORT.stl), the mold base (06-MOLD-BASE.stl), and the mold plugs (07-MOLD-PLUG.stl). Use the visual references in the middle panel of Fig. 3 to correctly position each part during the assembly process.

• **Step 1—**Begin by 3D printing the skull (01-SKULL.stl) and jaw (02-JAW.stl) using ABS with 100 % infill. As they are separate in a real human, print them separately, and remove any support structures. Connect the skull and the jaw using double-sided tape at the joint, and secure the mouth area with additional tape to simulate a closed position. This temporary setup will be stabilized by silicone rubber later. The complete skull assembly is shown in #1 in Fig. 4. Note: In our study, sanding or smoothing of the 3D-printed parts was not required, as the printed results were satisfactory.

### 5.2 Assembly of mold and skull

• **Step 4—**Insert four alignment pins into the triangular holes on one half of the mold, as shown in #3 of Fig. 4. These pins are used to ensure the proper alignment of the mold halves when they are assembled, also helping to maintain the correct positioning of the skull inside the mold.
• **Step 5—**Insert the supports into the designated holes on the front and back of the skull, then carefully align and position it within the mold, ensuring the supports fit securely into the corresponding slots, as shown in #4 of Fig. 4. Before positioning, seal any openings leading to the internal cavity (brain area) with clay to prevent leakage during the molding process.
• **Step 6—**Close the mold by connecting the alignment pins with the corresponding holes in the other half of the mold, see #5 of Fig. 4. Make sure both parts fit together to maintain the proper position.
• **Step 7—**Secure the mold base to its bottom section to prevent leaks during pouring, as shown in #6 of Fig. 4.
• **Step 8—**Seal any gaps in the mold with tape to prevent silicone leakage, and hold the mold tightly with three rubber bands, as shown in #7 of Fig. 4. The rubber bands help keep the mold halves together, preventing them from separating under the pressure of the silicone as it fills the mold.

### 5.3 Pouring and curing silicone

• **Step 9—** Mix silicone parts A and B in a plastic cup at a 1:1 ratio, using a 1-kg digital scale to accurately measure the weight (see #8 of Fig. 4). If desired, add pigment to achieve the preferred color, and mix thoroughly with a wooden stick. For ease, each cup should contain between 200–250 ml of material, as higher hardness levels can be more challenging to mix.
• **Step 10—**Pour approximately 6 cups of silicone into the mold from the top, as shown in #9 of Fig. 4. Fill it halfway, ensuring the material reaches the bottom of the internal structure, which can be monitored through the hole on top. This ensures proper coverage of the lower section, forming a secure base for the dummy head, as the supports need to be removed later.
• **Step 11—**Allow the silicone to cure, which typically takes 1 to 10 h, depending on the type used. Once the initial layer has solidified, remove the skull supports and replace them with the mold plugs, as shown in #10 of Fig. 4. The mold plugs share the same shape as the supports but are shorter in length. This ensures no holes remain in the front or back of the dummy head.
• **Step 12—**Continue filling the mold until it reaches the top of the skull, as shown in #11 of Fig. 4. Once filled, let it cure completely, which may take several hours.
• **Step 13—**After the silicone has fully cured, carefully remove the rubber bands and tape from the mold. Detach the mold plug and base, then gently separate the mold halves to reveal the dummy head, as shown in #12 of Fig. 4. Be cautious during this process to avoid damaging the mold, as it can be reused for making additional dummy heads.
• **Step 14—**Trim and clean any excess silicone from the edges of the dummy head to achieve a smooth finish, as illustrated in #13 of Fig. 4. Use scissors (or a sharp blade) to carefully remove any overflows around the surface.
• **Step 15—**Print the support stand (08-SUPPORT-STAND.stl) and install it under the dummy head, as shown in #14 of Fig. 4. The final completed dummy head with the support stand attached is shown in #15 of Fig. 4, ready for use in BC experiments.

## 6 Operation instructions

**Fig. 5:**
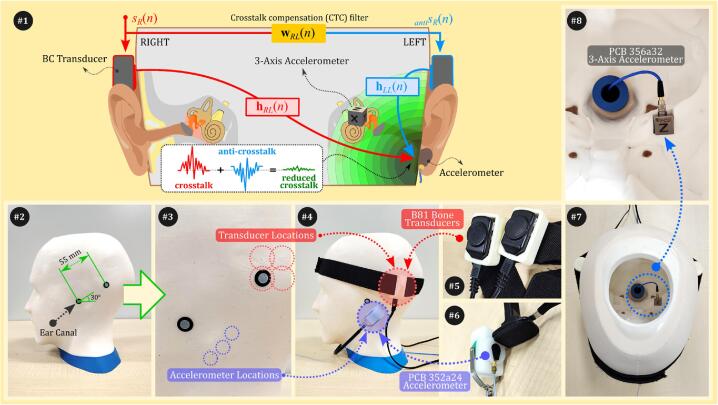
(#1) Illustration of an indirect method for crosstalk cancellation, targeting the mastoid with cancellation extending to the cochlea in the inner ear. (#2–8) Photos showing the setup on the dummy head: (#2) positioning of BC transducers at a specified angle from the ear canal, (#3) sensor locations, (#4) attachment details, (#5) close-up of B81 transducers, (#6) close-up of PCB 352a24 accelerometer, (#7) interior view of the dummy head showing the 3-axis accelerometer location, and (#8) close-up of PCB 356a32 3-axis accelerometer at the cochlea location.

The dummy head can be used in various BC experiments. But, finding the optimal combination of ABS and silicone hardness that closely matches human data is crucial and may vary depending on the specific experiment. Methods for BC crosstalk cancellation can be divided into two types: (i) psychoacoustic tests and (ii) adaptive algorithms with sensors [17], [18], [19], [20], [21]. Psychoacoustic tests [17], [18], [21] involve human subjects adjusting the phases of two pure tones to cancel crosstalk at the cochlea. While this method can be adapted for the dummy head by installing a 3-axis accelerometer at the cochlea location, it still relies on human feedback, making objective evaluation of the dummy head challenging. Thus, we focus on adaptive algorithms using an accelerometer on the mastoid [19] to indirectly cancel crosstalk sounds. In our previous study [19], as depicted in #1 of Fig. 5, we confirmed through experiments with seven human subjects that crosstalk cancellation at the mastoid extended to the cochlea. This approach is easier to implement on the dummy head as it allows for objective comparison by using the accelerometer as the target cancellation site. Therefore, in this section, we provide instructions for setting up the dummy head for this type of crosstalk cancellation, while details on algorithm implementation are available in our prior publication [19].

The instructions below outline the steps to set up “unilateral crosstalk cancellation” which targets the mastoid accelerometer. The cancellation effect is then extended to the cochlea, as depicted in #1 of Fig. 5. The following details how the dummy head was configured to explore this extension to the cochlea:

• Mark the dummy head to indicate the ear canal locations, see #2 of Fig. 5. These markings help accurately position the transducers since the dummy head does not have ears.
• Add additional markings approximately 55 mm from the ear canal, at the 2 or 10 o’clock positions. This corresponds to the BAHA location [29]. This step prepares the dummy head for transducer placement, as shown in #2 of Fig. 5.
• Attach the B81 bone transducers to the marked locations on the dummy head; see #4 of Fig. 5. Secure the transducers using a headband (#5 in Fig. 5) to ensure that they are properly positioned. Adjust the headband to apply a static force between 2.5 and 3.0 N [30]. This can be measured with a digital load cell temporarily placed between the transducer and the dummy head.
• Place a PCB 352a24 single-axis accelerometer inside a white soft silicone case and secure it using a RadioEar P-3333 steel band (#6 in Fig. 5). This setup ensures sufficient force for accurately capturing BC sounds. Position the PCB 352a24 accelerometer at the mastoid. This serves as the target cancellation site, as shown in #4 of Fig. 5.
• Because BC experiments can be sensitive to placement differences, we can repeat the experiment 12 times by slightly shifting the transducer and accelerometer between trials to account for positioning variability. Specifically, position the transducer at four different locations and the accelerometer at three locations on the mastoid, see #3 of Fig. 5.
• Place a PCB 356a32 3-axis accelerometer inside the dummy head at the cochlea location, as shown in #7 of Fig. 5. The choice of using 3-axis accelerometer is based on studies indicating that the cochlea is sensitive to vibrations from all directions [31]. The tri-axial cable connected to the accelerometer is routed through a hole in the support stand to ensure proper cable management; see #8 of Fig. 5.

**Fig. 6:**
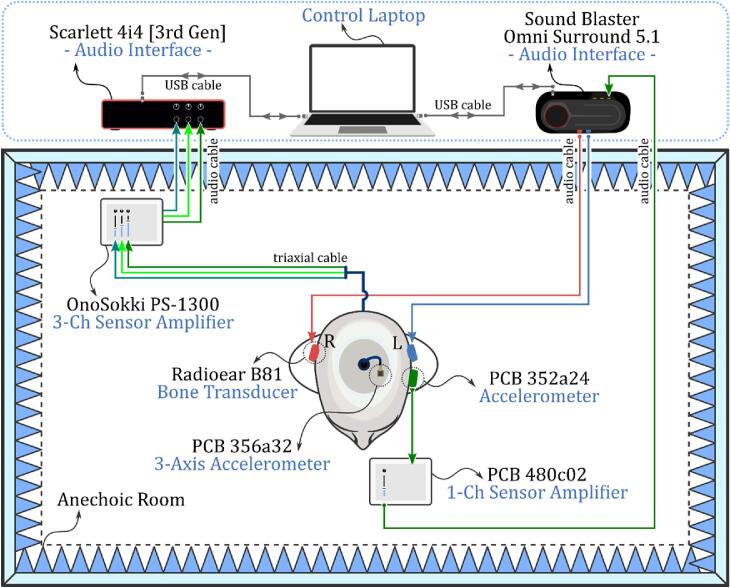
Audio setup used in the study, showing the connections between bone conduction transducers, accelerometers, sensor amplifiers, audio interfaces, and the control laptop.

The audio setup may vary depending on the available equipment. In this study, we retained the same setup as our previous work with human subjects [19], with the only differences being the addition of a 3-axis accelerometer and an additional audio interface. Fig. 6 illustrates the complete audio setup that shows all devices and their interconnections. The Sound Blaster Omni Surround 5.1 interface was used to send and receive signals to and from the B81 bone transducers and the PCB 352a24 accelerometer. Vibrational responses from the PCB 352a24 accelerometer were amplified using a PCB 480c02 1-Channel Sensor Amplifier before being recorded by the same interface. The Scarlett 4i4 [3rd Gen] interface was used exclusively to receive signals from the PCB 356a32 3-axis accelerometer at the cochlea, amplified via an OnoSokki PS-1300 Sensor Amplifier. All audio interfaces were controlled by a laptop, which handled both the playback of test signals and the recording of vibrational responses. Note that since the accelerometers are unaffected by air-conducted sounds, the experiment can also be conducted in a normal room.

This setup is the same as our previous experiments with human subjects [19]. In the dummy head, crosstalk cancellation at the cochlea is confirmed using the 3-axis accelerometer, whereas in human experiments, hearing threshold measurements are used for confirmation. This configuration facilitates easier comparisons between human and dummy head data, particularly in these parameters: (i) frequency responses from impulse responses $h_{\mathrm{RL}}\left(n\right)$ and $h_{\mathrm{LL}}\left(n\right)$, see #1 in Fig. 5; (ii) mastoid crosstalk cancellation at the accelerometer; and (iii) cancellation at the cochlea observed via the 3-axis accelerometer.

## 7 Validation and characterization

### 7.1 Purpose of validation

In this section, we aim to demonstrate the effectiveness and practicality of using 3D printing technology to create a skull with a silicone skin; this setup forms a dummy head to simulate crosstalk scenarios in BC. Our primary objective is to validate if this approach can replicate the conditions needed to study crosstalk cancellation. To do this, we compare the experimental results obtained from the dummy heads with those from our previous study involving human subjects. Although our previous study had a limited sample size of seven subjects, which may affect its generalizability, our focus here is different. We emphasize the flexibility and potential of this 3D-printed model. Specifically, we aim to show that by adjusting parameters like silicone skin hardness, we can fine-tune the dummy head’s response to closely match the data obtained from human subjects, demonstrating how adaptable the model can be.

### 7.2 Subjects and dummy heads

This study utilized four different dummy heads, each characterized by a different silicone skin hardness, while keeping a consistent skull material—ABS with 100 % infill. The silicone materials were purchased from SiliCreate (https://silicreate.com), and their hardness values were specified by the manufacturer. However, to ensure accuracy, we verified the actual hardness of each dummy head skin using a Shore 00 durometer (TECLOCK GS-754G) according to the ASTM D2240 standard. The hardness levels were found to range from 00–32 to 00–74. These were categorized as “Very Soft” (00–32), “Moderately Soft” (00–42), “Least Soft” (00–65), and “Slightly Hard” (00–74). For each dummy head, the experiment was repeated 12 times. Between trials, the transducer and accelerometer were slightly shifted to account for positioning variability. Specifically, the transducer was placed at four different locations, and the accelerometer at three locations on the mastoid, see #3 of Fig. 5. The averaged results from these repetitions were then analyzed.

The results from the dummy head experiments were then compared to data obtained from our previous study with human subjects aged between 21–32 years. While the experimental procedures were largely similar, the method for confirming crosstalk cancellation at the cochlea differed between the dummy heads and human subjects. For the dummy heads, a 3-axis accelerometer was placed at the cochlea location to measure BC sounds directly. These measurements were combined across the x, y, and z axes in the frequency domain using quadratic summation to calculate the total acceleration at each frequency [31]. The total acceleration $A_{\mathrm{tot}}$ is given by:

$$A_{\mathrm{tot}}^{2}=A_{x}∙A_{x}^{*}+A_{y}∙A_{y}^{*}+A_{z}∙A_{z}^{*}$$

where $A_{x}$, $A_{y}$, and $A_{z}$ are the accelerations in the x, y, and z directions, respectively, and ∗ denotes the complex conjugate. We computed the difference in total acceleration between conditions with and without the cancellation signal to quantify the effectiveness of the cancellation at the cochlea location.

In the human subjects, we measured their hearing thresholds using pure tones presented under narrowband noise centered at the test one-third octave band frequencies (250, 315, 397, 500, 630, 794, and 1000 Hz), both with and without the cancellation signal. These frequencies were selected to focus on the low-frequency range (≤1000 Hz), where cancellation method is most effective due to longer wavelengths. Additionally, using one-third octave band frequencies instead of standard octave bands provided more data points to evaluate cancellation performance across a denser range of frequencies. Successful cancellation of the band noise allowed the pure tone to be heard more clearly, resulting in lower hearing thresholds compared to the condition without cancellation. We then calculated the difference between these two thresholds to quantify the degree of cancellation at the cochlea.

## 8 Results and discussion

• *Frequency Response Comparison: Human vs. Dummy Heads*

**Fig. 7:**
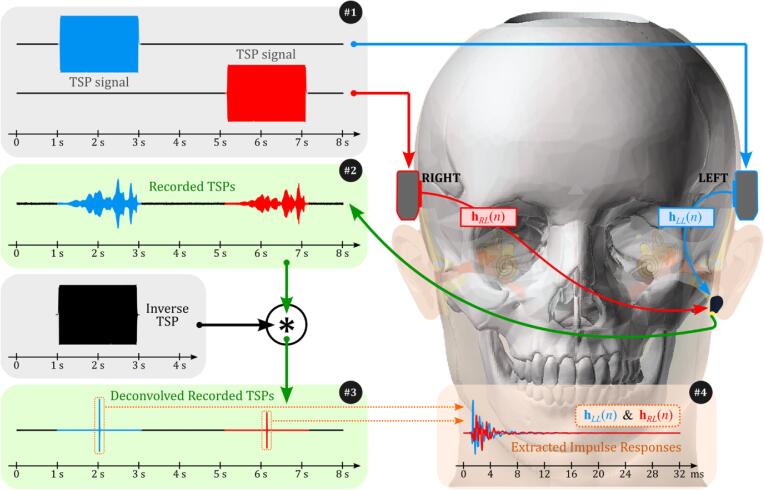
Illustration of the process for obtaining impulse responses from the dummy head. (#1) TSP signals for the left and right channels. (#2) Recorded TSP signals at the mastoid accelerometer. (#3) Deconvolution with inverse TSP signals to isolate the impulse responses. (#4) Final extracted impulse responses used for frequency response comparison between human and dummy heads.

In this study, we compared the frequency responses of dummy heads with different silicone skin hardness to human data to evaluate how well the silicone materials simulate BC sound transmission. All tests used a 3D-printed skull made of ABS, with silicone skin hardness as the only variable. Choosing the appropriate skin hardness is important to closely mimic human head responses. Fig. 7 illustrates the process for obtaining frequency responses from impulse response measurements. Here, $h_{\mathrm{RL}}\left(n\right)$ represents the impulse response from the right BC transducer to the accelerometer on the left mastoid, indicating the crosstalk path that needs cancellation. Meanwhile, $h_{\mathrm{LL}}\left(n\right)$ is the impulse response from the left BC transducer to the same accelerometer.

We began by generating time-stretched pulse (TSP) signals [32], arranged in stereo with signals in the left and right channels (see #1 of Fig. 7). These TSP signals were presented to the BC transducers attached to the dummy head. Vibrations resulting from BC sounds were recorded by an accelerometer on the mastoid (see #2 of Fig. 7). We then deconvolved these recorded signals with their inverse TSP signals to extract impulse responses, effectively isolating the system’s impulse response (see #3 of Fig. 7). The final impulse responses were extracted by selecting the first 32 ms of data, which captured the key characteristics of BC sound propagation within the dummy head, given the short distance between transducers and the accelerometer. These impulse responses are shown in #4 of Fig. 7.

**Fig. 8:**
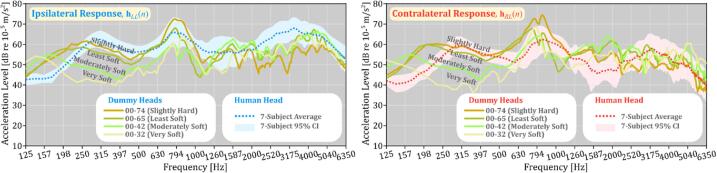
Frequency responses for ipsilateral (left) and contralateral (right) paths comparing dummy heads with four silicone hardness levels: 00–74 (Slightly Hard), 00–65 (Least Soft), 00–42 (Moderately Soft), and 00–32 (Very Soft) to human data. The human head data is represented by the 7-subject average (dotted line) and 95 % CI (shaded area).

The frequency responses were analyzed by applying FFT to the impulse responses $h_{\mathrm{LL}}\left(n\right)$ and $h_{\mathrm{RL}}\left(n\right)$: the ipsilateral response, where the transducer and the accelerometer on the mastoid are on the same side of the head, and the contralateral response, where the transducer is on the opposite side. The responses were summarized as averages from seven human subjects, with a 95 % confidence interval (CI) indicated by the shaded band. These are shown in Fig. 8, with the left panel displaying ipsilateral responses and the right panel showing contralateral responses. In the ipsilateral responses, harder silicone skins (00–74 and 00–65) resulted in higher acceleration levels, indicating more effective transmission from the ipsilateral transducer to the mastoid accelerometer. Particularly in the target cancellation frequency range (250–1000 Hz), the 00–74 (“Slightly Hard”) silicone skin provided responses very close to the 7-subject average and mostly within the 95 % CI. The 00–65 (“Least Soft”) skin also showed similar trends, though it slightly deviated from the 95 % CI in the 250–500 Hz range. In contrast, softer skins (00–42 and 00–32) produced responses that deviated more from human data, suggesting they are less effective in replicating human responses under the same conditions.

In the contralateral responses shown in the right panel of Fig. 8, the harder silicone skins (00–74 and 00–65) again provided results closest to the human data, with responses largely within the 95 % CI. However, at frequencies below 250 Hz, both ipsilateral and contralateral responses for the harder silicone skins were much higher than the human data. This difference may be due to the absence of a brain model inside the dummy head, resulting in a lower overall mass and, consequently, higher acceleration at the mastoid. The dummy head weighs approximately 2.75 kg, whereas the average mass of a human head is around 4.54 kg [33]. Despite these differences, the dummy heads with 00–74 and 00–65 silicone skins provided the closest match to human responses overall, suggesting their potential for simulating crosstalk scenarios.

• *Mastoid Crosstalk Cancellation: Comparative Effectiveness*

**Fig. 9:**
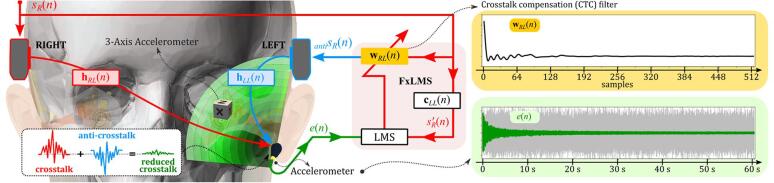
Illustration of the FxLMS algorithm for unilateral crosstalk cancellation. Impulse responses $h_{\mathrm{RL}}\left(n\right)$ and $h_{\mathrm{LL}}\left(n\right)$ simulate crosstalk, and the FxLMS adjusts the CTC filter coefficients $w_{\mathrm{RL}}\left(n\right)$ to minimize the error signal e(n) at the mastoid accelerometer, effectively generating an anti-crosstalk signal ${\;}_{\mathrm{anti}}s_{R}\left(n\right)$.

The frequency responses, particularly the contralateral response (see right panel of Fig. 8), reveal that a BC sound can travel from the opposite side of the head to the accelerometer on the mastoid, indicating crosstalk. Crosstalk occurs when sound from the contralateral (right) transducer reaches the mastoid but can be reduced by generating an anti-sound of equal amplitude and opposite phase from the ipsilateral (left) transducer (see Fig. 9). This anti-signal ${\;}_{\mathrm{anti}}s_{R}\left(n\right)$ is produced by passing the right input signal $s_{R}\left(n\right)$ through the crosstalk compensation (CTC) filter $w_{\mathrm{RL}}\left(n\right)$, with its coefficients estimated using the Filtered-x Least Mean Square (FxLMS) algorithm. Fig. 9 illustrates how the FxLMS algorithm and impulse responses obtained from the steps in Fig. 7 minimize the error signal e(n) at the mastoid accelerometer. Once e(n) converged (bottom right panel of Fig. 9), the final CTC filter coefficients $w_{\mathrm{RL}}\left(n\right)$ (top right panel of Fig. 9) were used to estimate the anti-crosstalk signal. This process mirrors the approach used in our previous human subject study.

**Fig. 10:**
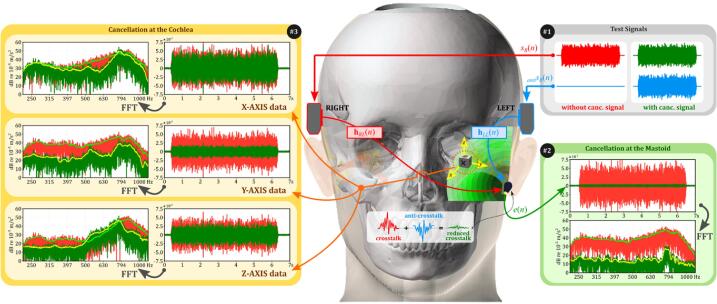
Procedure for evaluating crosstalk cancellation. Two filtered white noise signals (224–1122 Hz) were used (#1): one without (red) and one with (green) the cancellation signal, generated using the CTC filter (blue) from Fig. 8. These signals were played through BC transducers, with recordings taken at the mastoid to observe crosstalk reduction (#2). The analysis extended to the cochlea location, showing reduced vibrations across all axes, demonstrating that cancellation effects extended beyond the mastoid (#3). (For interpretation of the references to color in this figure legend, the reader is referred to the web version of this article.)

**Fig. 11:**
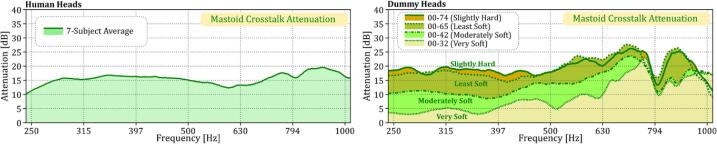
Mastoid crosstalk attenuation in decibels (dB) for dummy heads with varying silicone hardness (right) compared to the average attenuation for seven human subjects (left).

To evaluate the mastoid crosstalk cancellation performance, two 6-second filtered white noise signals (224–1122 Hz) were generated under two conditions: without (red) and with the corresponding cancellation signal (green), as shown in #1 of Fig. 10. The cancellation signal (blue) was obtained by passing the filtered noise through the previously determined CTC filter (Fig. 9). These two test signals were alternately played through the BC transducers attached to the dummy head, and the accelerometer on the mastoid recorded the resulting BC sounds. The recorded signals were then analyzed in both the time and frequency domains to assess cancellation effectiveness at various frequencies; see #2 of Fig. 10. This procedure was conducted on four dummy heads, and the difference between BC sound levels at the mastoid with and without the cancellation signal is expressed as “mastoid crosstalk attenuation” in decibels (dB), as shown in Fig. 11. The right panel of Fig. 11 shows attenuation levels for dummy heads with different silicone hardness levels, while the left panel displays the average crosstalk attenuation for seven human subjects.

In the human data shown in the left panel of Fig. 11, crosstalk attenuation varies between 10–20 dB, depending on the frequency. The dummy heads, however, display different levels of attenuation depending on the hardness of their silicone skins. The 00–74 (Slightly Hard) dummy head achieves attenuation levels from 14 to 26 dB, while the 00–65 (Least Soft) dummy head ranges from 16 to 28 dB. These results are relatively similar to the attenuation observed in human subjects, which is consistent with the fact that both 00–74 and 00–65 had frequency responses close to the human data, as seen in Fig. 8. In contrast, the Moderately Soft (00–42) and Very Soft (00–32) dummy heads show lower attenuation levels, especially below 500 Hz, reflecting their less accurate frequency responses in that range (see Fig. 8). The effectiveness of crosstalk cancellation—ranked from highest to lowest—follows the order of Slightly Hard, Least Soft, Moderately Soft, and Very Soft, which also aligns with the frequency response patterns observed in Fig. 8. Overall, while all dummy heads achieve some level of crosstalk cancellation at the mastoid, the degree of cancellation is linked to how closely their frequency responses match those of human subjects.

• *Crosstalk Cancellation at the Cochlea: Thresholds vs. Acceleration*

**Fig. 12:**
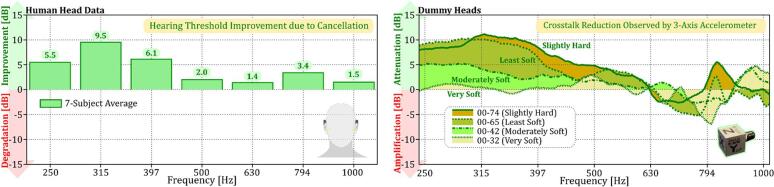
Crosstalk cancellation effectiveness at the cochlea in human subjects and dummy heads. The left panel shows average hearing threshold improvements in seven human subjects. The right panel displays crosstalk attenuation in dummy heads with varying silicone skin hardness, measured by a 3-axis accelerometer.

In this section, we aim to assess whether the crosstalk cancellation observed at the mastoid extends to the cochlea within dummy heads and to evaluate how these trends compare with observations in human subjects. For human subjects, hearing thresholds were measured under two conditions: without crosstalk cancellation, where narrowband noise centered at a test frequency was presented to the contralateral transducer and a pure tone to the ipsilateral transducer, and with crosstalk cancellation, where an additional anti-noise signal was introduced alongside the pure tone. Effective cancellation at the cochlea results in a lower hearing threshold compared to the non-cancellation condition, termed as “improvement,” while ineffective cancellation, resulting in the same or higher thresholds, is termed “degradation.” The observed “improvements” and “degradations” at each test frequency are shown in the left panel of Fig. 12.

For the dummy heads, crosstalk cancellation at the cochlea was evaluated using a 3-axis accelerometer, as shown in #3 of Fig. 10. Although the cancellation was primarily designed for the mastoid, its effects extended to the cochlea location, with varying performance across different frequencies and vibration directions (x, y, and z axes). To summarize these effects, we calculated the total acceleration differences across all three axes in the frequency domain, defining “attenuation” as successful cancellation and “amplification” as unsuccessful cancellation, as displayed in the right panel of Fig. 12.

When comparing the crosstalk cancellation at the mastoid (Fig. 11) to the cochlea (Fig. 12), we observe a decrease in effectiveness at the cochlea. Specifically, in human subjects, the crosstalk attenuation at the mastoid ranged from 10–20 dB (left panel of Fig. 11). However, when observing the improvement in hearing thresholds at the cochlea (left panel of Fig. 12), we found that this improvement significantly drops to between 1.4–9.5 dB. This indicates that while the cancellation does extend to the cochlea, the performance decreases notably, with the most significant improvements occurring at lower frequencies below 500 Hz, ranging from 5.5–9.5 dB. At higher frequencies between 500 and 1000 Hz, the improvement drops significantly, only ranging between 1.4–3.4 dB.

A similar trend in crosstalk cancellation at the cochlea is also observed in dummy heads with silicone skins of 00–74 (Slightly Hard) and 00–65 (Least Soft), which have frequency responses most similar to human subjects. As shown in the right panel of Fig. 12, crosstalk attenuation at frequencies below 500 Hz can reach up to approximately 11 dB. Similar to human data, at higher frequencies between 500–1000 Hz, the crosstalk cancellation performance drops; it can either attenuate or amplify depending on the frequency. This suggests that the cancellation does extend to the cochlea but is less effective at higher frequencies. In contrast, Moderately Soft (00–42) and Very Soft (00–32) dummy heads show lower reduction levels, likely because their baseline contralateral responses (right panel of Fig. 8) are already lower compared to other dummy heads and human data. This highlights the limitations of using softer silicone skins for accurately simulating crosstalk effects.

There are two possible reasons for the reduced crosstalk cancellation performance at the cochlea. First, the wavelength of the anti-sound signal plays a crucial role here. The cancellation signal is optimized for the mastoid with specific amplitude and phase. However, because the cochlea is some distance from the mastoid, the signal’s effectiveness decreases, especially at higher frequencies. Lower frequencies, with longer wavelengths, are less affected by distance. This explains the better performance observed at low frequencies. Second, the direction of vibrations also impacts cancellation as observed by the 3-axis accelerometer at the cochlea. As shown in Fig. 10, the single-axis accelerometer at the mastoid mainly measures movement in the y direction (see #2 of Fig. 10). For the same y-axis at the cochlea (see #3 of Fig. 10), crosstalk cancellation still occurs across all target frequencies, but at a lower level. The challenge comes from the x and z directions, where the cancellation is minimal or may even cause amplification. So, while y-axis reduction is effective at the cochlea, the overall acceleration—including x, y, and z axes—further reduces, leading to less crosstalk cancellation performance. Despite these challenges, dummy heads with harder silicone skins (00–74 and 00–65) show promise. They better match the hearing threshold improvements seen in human subjects, especially at lower frequencies. This indicates their potential for studying crosstalk cancellation in BC scenarios.

## 9 Summary

In this study, we focused on creating a dummy head using a 3D-printed skull and silicone skins to simulate bone conduction (BC) crosstalk scenarios. To evaluate the dummy head, we applied a crosstalk cancellation method from human studies, which used an accelerometer on the mastoid as the target cancellation site. By conducting similar experiments on the dummy heads, we were able to compare (i) frequency responses, (ii) crosstalk cancellation at the mastoid, and (iii) the extension of cancellation to the cochlea between human subjects and dummy heads. For the dummy heads, crosstalk effects at the cochlea were directly measured using a 3-axis accelerometer, while in human subjects, hearing thresholds were used for evaluation. We created four dummy heads with silicone skins categorized as Very Soft (00–32), Moderately Soft (00–42), Least Soft (00–65), and Slightly Hard (00–74) to determine which dummy head provided responses most similar to those observed in human subjects. The results showed that dummy heads with 00–74 and 00–65 hardness levels most closely matched human data across all three observations (i-iii). These findings demonstrate their potential to effectively simulate BC crosstalk scenarios. However, selecting the appropriate silicone hardness is essential, as it significantly affects how well the observations align with human data.

The applicability of the dummy head for BC studies could be enhanced by focusing future work on both physical improvements and computational enhancements. First, the total weight of the dummy head, which is only 2.75 kg, should be increased considering the average human head weight of 4.54 kg [33]. One way to achieve this is by adding a brain model made from a very soft material like silicone rubber Platsil-Gel 00–20 A + B [34] to contribute to the necessary mass adjustment. This material also can be tuned to have similar properties to the brain tissue in terms of tension and compression [34], [35]. Second, after achieving realistic weight distribution, the dummy head’s vibrational responses should be re-measured, and more human data should be collected to strengthen the baseline. Finally, signal processing techniques can be employed to iteratively refine the frequency responses to closely align with average human data.

## 10 Limitations

In this study, we treated our dummy head as a complete system, trying to find the best combination of an ABS skull and silicone skin of different hardness levels to mimic the vibrational responses observed in humans. However, this approach does not fully capture the unique properties of actual skulls and skin. Plus, our dummy head does not include a brain model, which might impact the vibrational responses if it were present. Also, we only looked at one head model, so we did not consider the variety in human skull sizes and shapes. This could limit how well our findings apply to real-world situations.

## 11 Ethics statements

This study included human participants, all of whom signed written informed consent forms. The research was carried out under protocol approved by the Institutional Review Board of Life Science Research at Chiba University.

## CRediT authorship contribution statement

**Irwansyah:** . **Sho Otsuka:** Writing – review & editing, Supervision, Resources. **Seiji Nakagawa:** Writing – review & editing, Supervision, Resources, Project administration, Funding acquisition.

## Declaration of competing interest

The authors declare that they have no known competing financial interests or personal relationships that could have appeared to influence the work reported in this paper.
